# Peptide Charge Derivatization as a Tool for Early Detection of Preeclampsia by Mass Spectrometry—A Comparison with the ELISA Test

**DOI:** 10.3390/molecules26237102

**Published:** 2021-11-24

**Authors:** Paulina Grocholska, Andrzej Konieczny, Zuzanna Kaźmierczak, Krystyna Dąbrowska, Karolina Panek-Laszczyńska, Marlena Kłak, Wojciech Witkiewicz, Zbigniew Szewczuk, Remigiusz Bąchor

**Affiliations:** 1 Faculty of Chemistry, University of Wroclaw, F. Joliot-Curie 14, 50-383 Wroclaw, Poland; paulina.grocholska@chem.uni.wroc.pl (P.G.); zbigniew.szewczuk@chem.uni.wroc.pl (Z.S.); 2 Department of Nephrology and Transplantation Medicine, Wroclaw Medical University, 50-556 Wroclaw, Poland; 3 Research and Development Center, Regional Specialized Hospital, 51-124 Wroclaw, Poland; zuzanna.kazmierczak@hirszfeld.pl (Z.K.); krystyna.dabrowska@wssk.wroc.pl (K.D.); marlena.klak@wssk.wroc.pl (M.K.); witkiewicz@wssk.wroc.pl (W.W.); 4 Immunology and Experimental Therapy, Polish Academy of Sciences, 53-114 Wroclaw, Poland; 5 Department of Obstetrics and Gynecology, Wroclaw Medical University, 50-368 Wroclaw, Poland; karolina.panek-laszczynska@umed.wroc.pl

**Keywords:** podocin, podocyturia, preeclampsia, charge derivatization, LC-MS, MRM, ELISA

## Abstract

Early detection of any preeclampsia biomarkers may lower the risk of mortality, both for a mother and a child. Our study focuses on techniques for preeclampsia biomarker identification by comparing the results of a method using liquid chromatography mass spectrometry in multiple reaction monitoring mode (LC-MS/MS) with those by the enzyme-linked immunosorbent assay (ELISA) test, as well as by comparing the obtained results with clinical data. In the proposed LC-MS/MS method a tryptic digest peptide charge derivatization strategy was used as a tool for sensitive detection of podocin, i.e., a previously discovered preeclampsia biomarker present in urine samples from pregnant women. Urine samples from pregnant women with diagnosed preeclampsia were collected at different stages of pregnancy and from healthy subjects, and then were analyzed by ELISA test and the proposed method with LC-MS/MS. Charge derivatization of the ε amino group of C-terminal lysine residues in tryptic digests by 2,4,6-triphenylpyrylium salt was performed to increase the ionization efficiency in the LC-MS/MS mode. Podocin was identified at the early stage of pregnancy, while its detection using an ELISA test was not possible. The protocol for urine sample preparation was optimized. Our results show that the proposed method by LC-MS/MS in combination with peptide charge derivatization, provides an ultrasensitive tool for diagnosis of preeclampsia, and provides earlier detection than a clinical diagnosis or ELISA test. The proposed solution may revolutionize medical diagnostics.

## 1. Introduction

Prevention is better than the cure; therefore, prediction of disease or a fast and accurate diagnosis is crucial for selecting the most effective therapy. New specific biomarkers are urgently needed to improve diagnostic methods; however, methods for the development and selection of reliable biomarkers are still challenging. Fortunately, they have been increasing steadily over the past decade [1]. In particular, robust biomarkers are those can be used to detect a disease at its early stage or predict a patient’s response to a therapy. Urine has always been one of the most promising body fluids, due to the non-invasive method of collection, and the fact that it consists of numerous molecular substances and several different biomarkers that can be analyzed simultaneously.

The detection of podocytes in urine is a promising diagnostic test suitable for early detection of kidney damage [2]. Podocytes are highly differentiated visceral epithelial cells that are involved in the maintenance of the glomerular filtration barrier. Pathological processes in glomeruli lead to podocyte detachment and subsequential excretion through the urinary tract; therefore, their presence may be detected in urine [3,4,5]. Podocytes are terminally differentiated cells with limited potential for regeneration, and therefore their loss is irreversible [6]. It has been proven that a 20–40% loss of glomerular podocytes, i.e., approximately 100–200 podocytes/glomeruli, led to denudation of the glomerular basement membrane (GBM) and consequently to glomerulosclerosis [7,8]. Numerous reports have indicated that early diagnosis of elevated urinary podocyte excretion can predict an acute phase of kidney injury and may facilitate the diagnosis of kidney disease in humans as well as animals [9]. A quantitative urinary protein analysis is strongly dependent on the degree of urine concentration [9,10]. Therefore, protein levels are assessed relative to the urine creatinine concentration: the urine podocin/creatinine ratio (UPoC), the urine albumin/creatinine ratio (UAC), and the urine protein/creatinine ratio (UPC) [10,11]. Therefore, results can be compared despite differences in the urine concentration, which is particularly significant in the case of patients with congestive heart failure (CHF) and chronic kidney disease (CKD), who have disturbances of urine dilution due to the disease itself or prescribed medication.

It has been proposed that more attention should be given to podocytes, a new marker of preeclampsia (PE) [12]. The presence of podocytes, as well as podocyte-specific proteins (such as podocin), in the urine of pregnant women, may predict PE at an early stage [13]. However, there is a very low abundance of these biomarkers in urine; therefore, there is an urgent need to develop a highly sensitive method for quantitative analysis of preeclampsia biomarkers, which is rapid, reliable, and can provide independent and confident results. Currently, immunoanalytical-based methodologies such as the enzyme-linked immunosorbent assay (ELISA) test, are often used to verify the presence of biomarkers in urine.

The immunoanalytical-based methods are considered to be the “gold standard” for protein quantification, since they are based on specific interactions between the protein of interest and a targeted antibody. Moreover, some immunoassays allow detection of extremely low amounts of proteins, in complex matrices such as plasma [14]. However, immunoassays rely on the availability of specific antibodies. In the case in which these antibodies do not exist, the production of antibodies is a time-consuming process, highly expensive, and the resulting antibodies do not always demonstrate a sufficient level of specificity to the targeted protein or may not discriminate protein isoforms, and as a result could under or overestimate protein levels [15].

According to data in the recent literature, a significant increase in targeted mass spectrometry method applications for biomarker analysis has been observed [16,17,18]. Mass spectrometry (MS)-based techniques do not require the use of antibody reagents, and therefore can be employed even when adequate antibodies are not available. MS can be used for precise identification of unknown analytes, quantification of compounds, and determination of structural and chemical properties of molecules. As compared with respected methods such as immunoassays, MS can be a method for rapid detection of multiple analytes with a level of specificity. Although tandem mass spectrometers are relatively expensive, the ability to perform up to hundreds of LC-MS/MS analyses per day, makes the price of a single analysis comparable to other modern medical diagnostics. Over the past two decades, the technology has become considerably more robust, accurate, and cost effective, leading to the steady adoption of MS in all subdisciplines of laboratory medicine. With increasing clinical applications of MS, there have been several reviews published on this topic, particularly focusing on clinical chemistry and more recently microbiology and hematology [19,20].

Garovic et al. [21] and Simon et al. [22] proposed MS-based methods for podocyturia detection in human urine samples, using tryptic digests of urine sediment samples and whole urine samples, respectively. Through their studies, they identified the new tryptic peptide fragments of podocin with the ^39^QEAGPEPSGSGR^50^ [21] and ^59^APAATVVDVDEVR^71^ [22] sequences, for the podocin quantification LC-MS/MS method.

In the last two years, we developed an LC-MS/MS method for the identification of podocin tryptic peptide in feline [23], canine [24], and equine [25] urine samples, using urine sediment samples obtained from animals with diagnosed CKD and acute kidney injury (AKI). We also revealed the advantage of mass spectrometry over the ELISA test, because of the higher sensitivity and selectivity of the MRM techniques [26].

Recently, we proposed a new and sensitive method for tryptic podocin peptide identification in urine sediments based on sample derivatization, using a fixed charge tag, in the form of 2,4,6-triphenylpyrylium salt and a targeted LC-MS/MS technique [27,28,29]. A peptide with the ^292^MIAAEAEK^299^ sequence, characteristic for both canonical and short podocin isoform, was identified in urine samples from patients diagnosed with PE, membranous nephropathy (MN), or IgA nephropathy (IgAN) both in native form and as a TPP conjugate. The study results revealed that the selected peptide was stable towards oxidation during the sample preparation steps.

In the presented study, we explored correlations in the clinical data of thirty-one pregnant women from a PE risk group, by comparing the data derived from ELISA tests and the proposed method with LC-MS/MS of urine sediment tryptic digest samples, used for the analysis of the presence of specific podocin-tryptic peptide. The aim of this study was to determine the accuracy of clinical tools for predicting PE development and the possibility of an additional application of the ELISA test and the LC-MS/MS methods, to improve efficiency. Additionally, we focused our attention on optimizing the protocol for urine sample preparation to avoid the loss of protein. We explored the applicability of a proposed method that combined peptide charge derivatization and LC-MS/MS as compared with the ELISA test for early detection of PE in urine sediment samples from pregnant woman. The proposed method may lower the risk of mortality, both for a mother and a child. To the best of our knowledge such a comparison has never been presented before.

## 2. Results

### 2.1. Patients

Thirty-one pregnant women, aged 31 ± 7 years, with at least one risk factor of preeclampsia, according to The American College of Obstetricians and Gynecologists (ACOG), were included into the prospective observational study. Three patients did not report to control visits. The follow-up time was 25 ± 4 weeks. The clinical characteristics are presented in Table 1.

Six of 28 patients (21%) developed gestational hypertension and 7 (25%) patients presented symptoms of PE. Patients who were affected by PE, presented higher values of blood pressure, especially in the 12th, 16th, 20th, 28th weeks of pregnancy. Data are presented in Table 2.

After implementing the logistic regression model, only one factor-systolic blood pressure at 20th week of pregnancy predicted PE (Table 3).

### 2.2. LC-MS/MS Analysis

The aim of this study was to investigate the applicability of the proposed method by LC-MS/MS combined with peptide charge derivatization as compared with the ELISA test for early detection of PE in urine sediment samples from women at different stages of pregnancy. Another goal of our investigation was to optimize the procedure of urine tryptic digest sample preparation. In our previous study [27], centrifugation using Amicon^®^ Ultra 10,000 MWCO Centrifugal Filters was the crucial part of the urine sample preparation, which allowed removal of DTT or CAM. During our study, we found that several washings of the urine sediment sample solution decreased the intensity of the signals corresponding to the analyzed MRM transitions. Therefore, we decided to optimize the urine sample preparation protocol by reducing the number of centrifugations and using an Amicon^®^ Ultra 10,000 MWCO filter. We observed that the best signal-to-noise ratios for the investigated MRM transitions were after each modification when centrifugation was not applied. Briefly, the optimized sample preparation procedure required pellet re-suspension in 1 mL of 0.1 M TEAB buffer containing Rapi-Gest™ SF (0.1% *v/v*) and sonication for 5 min, followed by the addition of 100 µL of 0.2 M DTT, and then the sample was incubated for 30 min at 60 °C. After that, 100 µL of 0.1 M CAM was added and the sample was incubated for 1 h at room temperature (in the dark). To the obtained sample, 50 μg of trypsin in 200 μL of 0.1 M TEAB was added, and the mixture was incubated at 37 °C overnight. The presented optimized protocol shortened the time of sample preparation and lowered the risk of protein loss. According to our previous study [27], the peptide with the ^292^MIAAEAEK^299^ sequence, characteristic for both canonical and short podocin isoform, was used as a podocin biomarker. To determine the detection sensitivity of the model peptide in the ESI-MS analysis, the level of its detection was analyzed using the LC-MS/MS mode. The following MRM transitions were analyzed: 862.45→347.20 (y_3_), 862.45→618.30 (y_6_), and 878.43→261.13 (b_2_). The most intense signal corresponded to the signal characterizing the y_6_ ion. The obtained data confirmed that the model peptide could be characterized at the femtomolar level of detection (12 × 10^−15^ mole) and the signal-to-noise ratio was 3:1. Previously, we proposed a peptide charge derivatization using 2,4,6-triphenylpyrylium salt, where the ε-amino group of lysine residue was modified, which resulted in the formation of 2,4,6-triphenylpyridinium salt containing a positive charge [28,29]. The formed positively charged tag increased the ionization efficiency in the MS-based analysis, lowering the detection limit of the compounds. Therefore, in the next step, the level of TPP-derivatized model peptide was analyzed in the LC-MS/MS mode where the following MRM transitions, selected automatically during the method of optimization, were monitored: [M + H]^2+^ 576.78→637.30 (y_3_) and 576.78→308.20 ([TPP + H]^+^). The most intense signal was the signal characterizing the y_3_ ion. The obtained data clearly indicate that the introduction of a fixed charge tag made it possible to analyze even 12 × 10^−18^ moles of compound when the signal-to-noise ratio was at least 3:1. To the best of our knowledge this is the most sensitive method of preeclampsia biomarker identification. The obtained curve describing the relationship between the amount of peptide sample introduced to the mass spectrometer and signal intensity had a linear character.

Additionally, as we proved previously [27], the *N*-terminal methionine residue in the used model peptide is stable towards oxidation during sample preparation and storage. The presence of the ^292^MIAAEAEK^299^ sequence in the form of TPP-derivative, as a podocin biomarker, was investigated in MRM analysis of the tryptic digests of urine sediment samples obtained from 31 pregnant women. We analyzed the samples at three different stages of pregnancy, i.e., 11th, 24th, and 36th week, to determine when the investigated biomarker could be identified by the proposed LC-MS/MS method. The samples were prepared according to the procedure described in the Section 4. The obtained data revealed the presence of podocin in the samples from seven subjects (22%). In the case of one pregnant woman (3%), the peptide with the ^292^MIAAEAEK^299^ sequence in the form of TPP-derivative was identified at the beginning, middle, and end of pregnancy. In one subject (3%) tryptic podocin peptide was identified at the middle of pregnancy and at the end. In six subjects the analyzed tryptic podocin peptide was identified at the end of pregnancy (19%). Four of the subjects with identified podocin were characterized by a DBP between 81 (mmHg) and 92 (mm Hg). Podocin was identified by LC-MS/MS in the samples of two subjects in their 4th pregnancy, four subjects in their 2nd pregnancy, and two subjects in their 1st pregnancy. The earliest detection of podocin was performed for a 44-year-old pregnant woman, 4th pregnancy, with a DBP of 83 (mmHg). For this patient, the ELISA test also revealed a significant increase in the podocin level of 63% between the first analysis and the last one. The tryptic podocin peptide was identified in the last analyzed sample of urine sediment from pregnant women, 26 years old, 1st pregnancy, with a DBP of 92 (mmHg).

**Figure 1:**
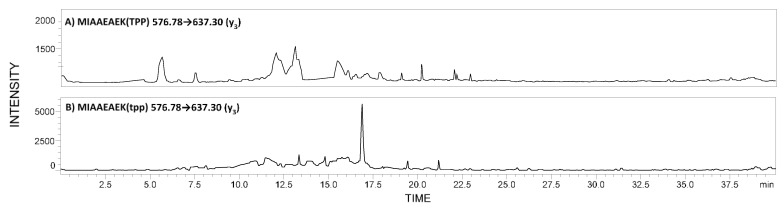
LC-MS/MS analysis of charge derivatized tryptic digests of urine sediment samples from: (**A**) A healthy pregnant women; (**B**) a subject with identified podocin. MRM transitions corresponding to the model peptide with the ^292^MIAAEAEK^299^ sequence.

The typical LC-MS/MS chromatograms presented selected MRM transitions obtained for the sample of a healthy subject (Figure 1A) and one subject with identified podocin (Figure 1B) are presented below.

In the case of the healthy subject, MRM transitions corresponding to the peptide with the ^292^MIAAEAEK^299^ sequence derivatized by TPP ionization tag are absent (Figure 1A). Although, Vogelmann and co-workers [10] suggested the possibility of podocyte excretion in healthy subjects, the small amounts of released podocytes and podocin make their identification impossible. Additionally, Garovic et al. [30] reported a lack of podocytes in urine samples from healthy pregnant women.

### 2.3. ELISA Test

The ELISA test was performed for 28 samples from different pregnant women (Table 4). Positive ELISA tests were noted for 8 pregnant women. In the same subjects, we also identified the presence of tryptic podocin peptide by the proposed LC-MS/MS method. The podocin levels as detected in urine by ELISA were very low (close to detection limit) and highly variable. No significant correlations with medical symptoms nor consistency between samples were observed. For the patient with the earlier diagnosis of podocin by LC-MS/MS (a 44-year-old woman, 4th pregnancy, with a DBP of 83 (mmHg)) the ELISA test revealed a significant increase in podocin level of 63% between the first analysis and the last one.

## 3. Discussion

A comparison between the applicability of the clinical data, including systolic and diastolic blood pressures, for predicting preeclampsia, with podocin identification using the ELISA test and the proposed LC-MS/MS method, has never been described before. Therefore, in this study, we decided to investigate which of the presented methods could detect podocin, as podocyturia and preeclampsia biomarkers, in the early stage of pregnancy. Additionally, we compared all obtained data from each applied method.

In 2013, Garovic et al. [21] and Simon et al. [22] presented the possibility of applying LC-MS/MS for podocyte detection using a qualitative and quantitative approach. The proposed strategy was successfully applied in the analysis of tryptic peptide of podocin containing arginine residue at the C-terminus. It is known that peptides containing arginine residue, which is the most basic amino acid, produce a higher abundance of ions in MS spectra than peptides containing lysine or other residues. Consequently, peptides containing arginine produced by tryptic digestion of proteins are the most abundant in mass spectra [26]. Lysine-containing peptides are usually not conceded as good biomarkers due to their low ionization efficiency in MS experiments. However, those peptides may serve as a potential biomarker of some diseases, and therefore development of a method for their identification is an important task which was the aim of this study. Recently, there has been significant interest in proteomic investigations to develop methods for chemical sample modification which increases the sensitivity of the mass spectrometry analysis of tryptic peptides, using their derivatization with ionization enhancers containing a fixed positive charge resulting from the chemical structure of a derivatizing agent.

A performed LC-MS/MS study revealed the presence of tryptic podocin peptide in the urine sediment samples from seven subjects. In the case of six of the patients, their pregnancies were terminated by cesarean sections. In one podocin-positive subject where the peptide with the MIAAEAEK was identified at the end of the pregnancy, the pregnancy was terminated by vaginal birth. The results obtained in this study clearly confirmed that the application of a fixed charge tag in the form of 2,4,6-triphenylpyrylium salt makes the ultrasensitive LC-MS/MS analysis of lysine-containing peptide possible. The tryptic podocin peptide used in our study has a methionine residue at the *N*-terminus which, according to the selection criteria for proteomic analysis reported by Mohammed et al. [31], should be avoided due to the possibility of oxidation to final sulfoxide resulting in the 16 Da mass shift disrupting the sensitivity of detection and quantification by mass spectrometry. As we presented before [27], the selected peptide with the MIAAEAEK sequence does not undergo oxidation during the sample preparation making its application in the LC-MS/MS analysis possible. Additionally, this peptide is characteristic for both canonical and short podocin isoform [32,33], which may also play different physiological roles [34].

According to the clinical definition, preeclampsia is characterized by new onset or worsening hypertension (SBP ≥ 140 mmHg or DBP ≥ 90 mmHg) and proteinuria (greater than or equal to 300 mg/24 h urine collection or protein/creatinine ratio ≥ 0.3). In the absence of proteinuria, other symptoms may indicate preeclampsia including thrombocytopenia, impaired liver function, pulmonary oedema, and new onset visual or cerebral disturbance [35,36]. Recently, Bullarbo and Rylander [37] demonstrated the utility of an increase in diastolic blood pressure as a useful tool for early identification of preeclampsia. The presented results suggested that a base-line diastolic blood pressure of ≥80 mmHg and an increase in diastolic blood pressure of ≥15 mmHg may be useful in the identification of women at risk of preeclampsia (sensitivity 92%, specificity 44%) and as a selection criterion in treatment or prevention assays. In our study, we found a correlation between the presence of podocin identified by LC-MS/MS and the ELISA test and higher levels of DBP (Table 4). In our study, we found that the amount of podocin in human urine samples determined by the ELISA test was very low. Our findings indicate that the ELISA test is not a sufficient method for assessing changes in the level of podocin in human urine samples. To detect the levels of podocin, signal amplification (e.g., by chemical methods) must be applied.

First of all, clinical parameters such as blood pressure, serum aminotransferases, creatinine concentration, and proteinuria (as assessed by UPCR) were taken into consideration. Blood pressure values differed only between those women who developed preeclampsia and those who did not. After implementation of logistic regression, only diastolic blood pressure, at the 20th week of pregnancy (OR 1.2, CI 1.02–1.42, *p* = 0.025), predicted occurrence of PE (Table 5, Table 6 and Table 7).

In contrast, urinary podocin concentrations, assessed by the ELISA test, did not differ significantly between both groups. We also measured podocin signal using the the proposed LC-MS method at three time points, i.e., the 11th, 24th, and 36th week of pregnancy. A higher positive predictive value and accuracy were achieved during the 24th week of pregnancy. We were able to show the superiority of the proposed LC-MS method as compared with the ELISA test measurements.

The proposed method could identify the tryptic podocin peptide even during the early stages of pregnancy where proteinuria, commonly related to the podocyturia, was not identified by clinical diagnosis [37,38]. The specificity of proteinuria has been questioned in view of the poor relation between the 24 h excretion and spot check data [39]. The most important factor in the discussion of the obtained results is the correlation of the results obtained by the proposed LC-MS/MS method and the ELISA test with DBP values.

## 4. Materials and Methods

### 4.1. Chemicals

All the solvents and reagents were used as supplied. Fmoc amino acid derivatives and the Fmoc-Lys(Mtt)-Wang resin (0.56 mmol/g) were purchased from Novabiochem (Darmstadt, Germany). *N*-[(dimethylamino)-1*H*-1,2,3-triazolo-[4,5-b]pyridin-1- ylmethylene]-*N*-methylmethanaminium hexafluorophosphate *N*-oxide (HATU) and trifluoroacetic acid (TFA) were obtained from IrisBiotech (Marktredwitz, Niemcy). Solvents for peptide synthesis (*N,N*-dimethylformamide (DMF); dichloromethane (DCM); *N*-ethyldiisopropylamine (DIEA); tetraethylammonium bicarbonate (TEAB); *N,N,N*-triethylamine; 1,2-dithiotreitol (DTT); H_2_O_2_; tetrafluoroborate 2,4,6-triphenylpyrylium salt; and iodoacetamide were obtained from Sigma-Aldrich (Saint Louis, MO, USA). Triisopropylsilane (TIS) was purchased from Fluka (Bucharest, Romania). Amicon^®^ Ultra 10,000 MWCO Centrifugal Filters were purchased from Merck (Darmstadt, Germany), Rapi-Gest™ SF was from Waters (Waters Chromatography Europe BV, Etten-Leur, The Netherlands).

### 4.2. Peptide Synthesis

The synthesis of the model peptide on the Fmoc-Lys(Boc)-Wang resin was performed manually in polypropylene syringe reactors (Intavis AG) equipped with polyethylene filters, according to a standard Fmoc (9-fluorenylmethoxycarbonyl) solid phase synthesis procedure [40].

### 4.3. In Solution Peptide Derivatization with 2,4,6-Triphenypyrilium Salt

The purified model peptides (0.2 mg) were dissolved, in a separate Eppendorf tube, in 0.5 mL of DMF containing 0.05 mg of 2,4,6-triphenylpyrylium tetrafluoroborate (TPP) and 19 μL of *N,N,N*-triethylamine was added. The mixture was incubated at 60 °C for 1 h and the solution was evaporated under a nitrogen stream. The final product was dissolved in 15% MeCN/H_2_O mixture (*v/v*), lyophilized, and used for MS/MS analysis and MRM method development.

### 4.4. Purification

The synthesized peptide was purified using an analytical HPLC Thermo Separation system with UV detection (210 nm) with a YMC-Pack RP C18 column (4.6 × 250 mm, 5 μm), with a gradient elution of 0–40% B in A (A = 0.1% TFA in water and B = 0.1% TFA in acetonitrile/H_2_O, 4:1 *v/v*) over 30 min (flow rate 1 mL/min). The main fraction, corresponding to the peptide, was collected and lyophilized.

### 4.5. Mass Spectrometry

The ESI-MS experiments were all performed on a LCMS-9030 qTOF Shimadzu (Shimadzu, Kyoto, Japan) device, equipped with a standard ESI source and the Nexera X2 system. The analysis was performed in the positive ion mode between 50 and 2000 *m/z*. The LCMS-9030 parameters were as follows: nebulizing gas, nitrogen; nebulizing gas flow, 3.0 L/min; drying gas flow, 10 L/min; heating gas flow, 10 L/min; interface temperature, 300 °C; desolvation line temperature, 400 °C; detector voltage, 2.02 kV; interface voltage, 4.0 kV; collision gas, argon; collision energy was optimized between 10 and 30 eV. The injection volume was 5 μL. The obtained signals all had a mass accuracy error in the range of 1 ppm. The used solvents were all of LC-MS grade. The chromatographic module was operated as follows: eluent (A) water + 0.1% HCOOH, eluent (B) acetonitrile + 0.1% HCOOH. The obtained data were analyzed by LabSolutions software (Shimadzu, Kyoto, Japan).

### 4.6. Urine Sample Collection and Preparation

Pregnant women with at least one risk factor of PE were recruited into the observational prospective study. The following risk factors were considered: a PE history, age >40 years, a family history of PE, chronic hypertension, chronic renal disease (CKD), autoimmune disease (e. g. antiphospholipid syndrome (APS), systemic lupus erythematosus (SLE)), pregestational diabetes mellitus, multifetal gestation, obesity, nulliparity, use of assisted reproductive technology.

Two primary end points were estimated: appearance of gestational hypertension and/or preeclampsia. Gestational hypertension was defined as a new onset of hypertension (established as systolic blood pressure (SBP) ≥ 140 mmHg and/or diastolic blood pressure ≥ 90 mmHg) at ≥ the 20th week of gestation, documented on at least two visits. At each appointment, blood pressure was measured twice, 10 min apart. Preeclampsia was characterized as gestational hypertension, with 1 or more of the following: proteinuria defined as urine protein to creatinine ration (UPCR) ≥ 0.3 g/g, platelet count < 100,000/μL, serum creatinine > 1.1 mg/dl or doubling of creatine concentration in the absence of renal disease, liver transaminases at least twice the upper limit, pulmonary edema, new onset and persistent headache, and visual symptoms (blurred vision, flashing lights, or sparks). Preeclampsia was considered to be superimposed when it occurred in a woman with chronic hypertension. It was characterized by worsening or resistant hypertension, new onset of proteinuria, or sudden increase of proteinuria, after 20 weeks of gestation in a woman with chronic hypertension. During the follow-up, control visits were scheduled every 4 weeks until the 32nd week of gestation and every 2 weeks after the 32nd week of gestation. At every visit, blood pressure was measured twice, 15 min apart. Blood samples and first voided urine (collected at most in 1 h) were collected at each appointment. The following parameters were assessed: complete blood count (CBC), serum concentrations of alanine transaminase (ALT) and aspartate transaminase (AST), serum creatinine concentration (sCr), urinary protein and creatinine concentrations. Urinary protein to creatinine ratio (UPCR) was calculated by dividing urine protein concentration (mg/dl) and urinary creatinine concentration (mg/dl). The optimized procedure for the urine sample preparation for comparison with that previously presented by [27] was applied. Collected urine samples (~1 mL each) were centrifuged for 10 min at 5000 rpm at 30 °C. The supernatant was discarded, and the pellet was resuspended in 1 mL of 0.1 M TEAB buffer containing Rapi-Gest™ SF at a concentration of 0.1% and sonicated for 5 min. In the next step, 100 µL of 0.2 M DTT was added, the sample was incubated for 1 h at 30 °C. After that, 100 µL of 0.1 M CAM was added and the sample was incubated for 1 h at room temperature (in the dark). Then, a sample of 50 μg of trypsin in 200 μL of 0.1 M TEAB was added and the sample was incubated at 37 °C overnight. After digestion, 20 μL of formic acid was added and the sample was lyophilized. The dry solid was dissolved in 15% MeCN/H_2_O and used for the LC-MS analysis.

### 4.7. Urine Tryptic Digest Derivatization with 2,4,6-Triphenypyrilium Salt

The lyophilized tryptic urine hydrolates were dissolved in 0.5 mL of DMF solution containing 0.25 mg of 2,4,6-triphenylpyrylium tetrafluoroborate and 95 μL of *N,N,N*-triethylamine. The mixture was incubated at 60 °C for 1 h and the solution was evaporated under a nitrogen stream. The final product was dissolved in 15% MeCN/H_2_O mixture (*v/v*) and lyophilized.

### 4.8. Liquid Chromatography Mass Spectrometry (LC-MS) Analysis in Multiple Reaction Monitoring (MRM) Mode

LC-MS/MRM experiments were performed on a LCMS-8050 Shimadzu apparatus, with a UHPLC Nexera X2 system, equipped with an Aeris Peptide XB-C18 column (50 mm × 2.1 mm) 3.6 μm bead diameter, equilibrated at 27 °C. The LC system was operated with mobile phase, consisting of solvent A (0.1% formic acid in H_2_O) and solvent B (0.1% formic acid in MeCN). The gradient conditions (B%) were from 5 to 80% B within 40 min. The flow rate was 0.1 mL/min, and the injection volume was 5 μL. The MRM method was optimized automatically, and the following transitions were chosen for ^292^MIAAEAEK^299^ 862.45→347.20 (y_3_) and 862.45→547.28 (y_5_); for MIAAEAEK(TPP) [M + H]^2+^, 576.78→637.30 (y_3_) and 576.78→308.20 ([TPP − H]^+^).

### 4.9. Statistical Analysis

The statistical analysis was performed using Statistica ver. 13 software (StatSoft, Tulsa, OK, USA). The quantitative continuous data were expressed as the mean and standard deviation (SD). The distribution was tested with the use of the Kolmogorov–Smirnov test. Differences between the two groups were assessed with the use of a *t*-test. Categorical variables were expressed as absolute (N) and percentage (%) values and compared using the χ^2^ test. To predict the occurrence of preeclampsia, a logistic regression model was built. A *p*-value < 0.05 was considered to be statistically significant.

### 4.10. Podocin Detection in Human Urine Samples by ELISA

The levels of podocin in human samples were assessed by the ELISA test. The pellet of the collected urine samples was resuspended in 1 mL of Denaturating Cell Extraction Buffer (Invitrogen cat. no. FNN0091). The samples were incubated on ice for 30 min, and then centrifuged 10,000× *g* for 15 min. Samples were used to measure podocin (pg/mL) levels using commercially available ELISA (Human podocin ELISA Kit cat. no. 201-12-1083, SunRed Biotechnology Company, Shanghai, China), according to the manufacturer’s instructions. Then, 100 µL of each sample was added to the microwell plate pre-coated with an antibody specific to human podocin and incubated at 37 °C for 2 h. The liquid from each well was removed, and Detection Reagent A was added. The plate was incubated at 37 °C for 1 h. Then, wells were washed three times, and the Detection Reagent B was added. The plate was incubated at 37 °C for 1 h. The plate was washed five times, and the substrate solution was added, 100 μL/each well. The plate was incubated at 37 °C for 20 min in the dark. At the end of incubation, 50 μL stop solution was added to each well. Absorbance was assessed with an absorbance microplate reader (PowerWave HT, BioTek Instruments, Bad Friedrichshall, Germany). The absorbance was measured at 450 nm, and a four-parameter curve fitting was employed to calculate the analyte concentration. All measurements were performed in duplicate.

## 5. Conclusions

In conclusion, here, we have presented the advantage of a proposed method by LC-MS/MS over the ELISA test, as well as clinical diagnosis, for early identification of PE, in the tryptic digests of urine sediment samples obtained from pregnant women. The applied podocin tryptic peptide with the ^292^MIAAEAEK^299^ sequence, derivatized using 2,4,6-triphenylpyrylium fixed charge tag, was successfully used in the qualitative identification of podocin, making ultrasensitive detection even at the attomolar level by LC-MS/MS possible. Although, the results obtained by the ELISA test were confirmed by MS analysis in some cases, we found that the ELISA test was not as sensitive as the proposed method by LC-MS/MS. Additionally, the podocin-positive samples identified using the proposed method with LC-MS/MS were characterized by higher values of DBP. The proposed strategy may facilitate early identification of women at risk of developing preeclampsia using targeted, antenatal surveillance and may provide a tool for intervention.

## Figures and Tables

**Table 1:** Baseline characteristics. Abbreviations: BMI, body mass index; SBP, systolic blood pressure; DBP, diastolic blood pressure; PE, preeclampsia; CKD, chronic kidney disease.

Variable	Baseline Values
Age (years)	31 ± 7
Weight (kg)	73 ± 15
Height (cm)	168 ± 5
BMI (kg/m^2^)	26 ± 5.5
SBP (mmHg)	127 ± 13
DBP (mmHg)	79 ± 8
Gestational week at qualification	12 ± 2
Gestational week at the time of delivery	37 ± 4
Elective cesarean delivery	12/28 (43%)
Emergency cesarean section	13/28 (46%)
Vaginal birth	3/28 (11%)
1st pregnancy	12/31 (39%)
2nd pregnancy	13/31 (42%)
3rd pregnancy	4/31 (13%)
4th pregnancy	2/31 (6%)
Multiple pregnancy	4/31 (13%)
A past history of PE	13/31 (42%)
Chronic hypertension	9/31 (29%)
CKD	2/31 (6%)
A family history of PE	1/31 (3%)
Pregestational diabetes	2/31 (6%)

**Table 2:** Significant differences in blood pressure among patients who were affected by PE. Abbreviations: SBP, systolic blood pressure; DBP, diastolic blood pressure; PW, pregnancy week.

Variable	Preeclampsia		*p*-Value
	YES	NO	
SBP 12th PW (mmHg)	134 ± 13	123 ± 11	0.049
BDP 12th PW (mmHg)	84 ± 7	76 ± 8	0.035
DBP 16th PW (mmHg)	83 ± 4	73 ± 9	0.016
SBP 20th PW (mmHg)	131 ± 8	119 ± 12	0.024
DBP 20th PW (mmHg)	86 ± 5	73 ± 9	0.004
SBP 28th PW (mmHg)	136 ± 17	123 ± 10	0.03
DBP 28th PW (mmHg)	86 ± 11	76 ± 8	0.03
SBP 32th PW (mmHg)	131 ± 14	122 ± 8	0.046

**Table 3:** Logistic regression results for predicting PE. OR—odds ratio, CI—confidence interval.

Variable	Estimate	OR	CI	*p*-Value
SBP at 20th week of pregnancy	0.18	1.2	1.02–1.43	0.025

**Table 4:** Comparison of the clinical LC-MS/MS and ELISA data for the diagnosis of preeclampsia. Levels of podocin in human urine samples based on the ELISA test. Results were normalized and values were calculated regarding a standard curve, obtained for podocin-coated wells. “Undetectable” means a reading below the detection level; PE, preeclampsia; “+”, positive result; “-”, negative result.

Patient No.	Podocin Level at the Beginning of Pregnancy (ng/mL)	Podocin Level at the End of Pregnancy (ng/mL)	% Change
1	1.166	0.473	−59
2	0.321	0.878	174
3	2.128	1.588	−25
4	0.625	0.693	11
5	0.49	0.895	83
6	1.318	0.997	−24
7	Undetectable	0.659	
8	1.301	0.439	−66
9	0.051	1.402	2649
10	0.591	0.659	12
11	Undetectable	0.608	
12	0.895	0.321	−64
13	Undetectable	0.017	
14	0.253	-	
15	Undetectable	0.321	
16	0.287	1.334	365
17	0.051	0.321	529
18	Undetectable	0.152	
19	Undetectable	Undetectable	
20	0.27	0.101	−63
21	0.338	6.976	1964
22	0.321	Undetectable	
23	1.081	0.22	−80
24	0.659	0.811	23
25	0.726	0.405	−44
26	0.186	0.118	−37
27	Undetectable	Undetectable	
28	1.284	Undetectable	

**Table 5:** Statistical analysis of preeclampsia prediction using the proposed LC-MS/MS method in 11th week of pregnancy. CI—confidence interval.

Statistic	Value	95% CI
Sensitivity	14.29%	From 0.36 to 57.87%
Specificity	100.00%	From 83.89 to 100.00%
Positive likelihood ratio		
Negative likelihood ratio	0.86	From 0.63 to 1.16
Disease prevalence	25.00%	From 10.69 to 44.87%
Positive predictive value	100.00%	
Negative predictive value	77.78%	From 72.12 to 82.57%
Accuracy	78.57%	From 59.05 to 91.70%

**Table 6:** Statistical analysis of preeclampsia prediction using the proposed LC-MS/MS method in the 24th week of pregnancy. CI—confidence interval.

Statistic	Value	95% CI
Sensitivity	85.71%	From 42.13 to 99.64%
Specificity	100.00%	From 83.89 to 100.00%
Positive likelihood ratio		
Negative likelihood ratio	0.14	From 0.02 to 0.88
Disease prevalence	25.00%	From 10.69 to 44.87%
Positive predictive value	100.00%	
Negative predictive value	95.45%	From 77.38 to 99.23%
Accuracy	96.43%	From 81.65 to 99.91%

**Table 7:** Statistical analysis of preeclampsia prediction using the proposed LC-MS/MS method in the 36th week of pregnancy. CI—confidence interval.

Statistic	Value	95% CI
Sensitivity	85.71%	From 42.13 to 99.64%
Specificity	90.48%	From 69.62 to 98.83%
Positive likelihood ratio	9.00	From 2.33 to 34.80
Negative likelihood ratio	0.16	From 0.03 to 0.97
Disease prevalence	25.00%	From 10.69 to 44.87%
Positive predictive value	75.00%	From 43.69 to 92.06%
Negative predictive value	95.00%	From 75.48 to 99.15%
Accuracy	89.29%	From 71.77 to 97.73%

## Data Availability

Not applicable.

## Sample Availability

Samples of the compounds are not available from the authors.
